# On-chip microwave oscillator in deep-subwavelength scale based on spoof plasmonic skyrmions

**DOI:** 10.1093/nsr/nwag028

**Published:** 2026-01-19

**Authors:** Wan Zhu Wang, Yu Liu, Xuanru Zhang, Zhi Lin Gao, Xu Sheng Tang, Tie Jun Cui

**Affiliations:** State Key Laboratory of Millimeter Waves, Southeast University, Nanjing 210096, China; School of Information Science and Engineering, Southeast University, Nanjing 210096, China; State Key Laboratory of Millimeter Waves, Southeast University, Nanjing 210096, China; School of Information Science and Engineering, Southeast University, Nanjing 210096, China; State Key Laboratory of Millimeter Waves, Southeast University, Nanjing 210096, China; School of Information Science and Engineering, Southeast University, Nanjing 210096, China; State Key Laboratory of Millimeter Waves, Southeast University, Nanjing 210096, China; School of Information Science and Engineering, Southeast University, Nanjing 210096, China; School of Cyber Science and Engineering, Southeast University, Nanjing 210096, China; State Key Laboratory of Millimeter Waves, Southeast University, Nanjing 210096, China; School of Information Science and Engineering, Southeast University, Nanjing 210096, China

**Keywords:** deep subwavelength, microwave oscillator, skyrmions, spoof localized surface plasmons, CMOS chip

## Abstract

Exploring wave-matter interactions at extreme electromagnetic (EM) scales has long been a focal topic in multidisciplinary research, but deep-subwavelength field localization remains a persistent challenge. Here, we demonstrate an on-chip microwave oscillator with an electrical size of *λ*_0_/865 (*λ*_0_ is the operating wavelength) and a mode volume of 2.97 × 10^−11^ *λ*_0_^3^ using the 0.18 μm complementary metal-oxide-semiconductor (CMOS) process. The physical insight is based on a spoof plasmonic skyrmion resonator realized by square spiral metal lines, which simultaneously functions as a frequency selector and an energy storage element for EM signal generation. A strongly-coupled excitation structure is proposed to efficiently concentrate EM energy into the deep-subwavelength resonance structure, and a complementary cross-coupled pair is employed to compensate for the high material loss resulting from the extreme energy concentration. This work paves the way for integrating the skyrmion resonances into the CMOS chips in extremely deep-subwavelength scale, and holds great promise for miniaturized integrated circuits and systems.

## Teaser

This work presents the pioneering integration of electromagnetic skyrmions with active circuits in the 0.18 μm CMOS process, achieving a deep-subwavelength on-chip microwave oscillator.

## INTRODUCTION

The study of wave-matter interactions in extreme electromagnetic (EM) dimensions has been a cutting-edge topic in multiple disciplines since the inventions of the maser in 1954 [1] and the laser in 1960 [2]. Strongly localized EM fields in frequency, time, momentum, and space domains provide powerful tools to investigate the limits of EM physics [3–6]. Spatially localized EM fields have emerged as research hotspots, including optical singularities, plasmonic hotspots, and nano-lasers [6–8]. Constrained by the diffraction limit, the key challenge in subwavelength wave generation lies in achieving an optimal balance between the size and performance of the filtering resonance. At optical frequencies, surface plasmons arising from electron density oscillations on metal-dielectric interfaces can provide enhanced momentum and effectively confine the mode volume [9,10]. Hence, plasmonic lasers with elaborately designed plasmonic resonance structures emerged as promising techniques, leveraging plasmonic nanoparticles [11], hybrid plasmonic waveguides [12], and other advanced configurations [13]. Based on sharp-tip and nano-gap effects, dielectric bowtie nano-antennas can confine the optical fields to an atomic-scale mode volume, with a reported laser volume as low as 0.0005 *λ*_0_^3^, where *λ*_0_ is the operating wavelength [6]. At microwave frequencies, managing electrical sizes becomes even more challenging due to the considerably larger wavelengths [14,15]. Though lumped inductors and capacitors can be used, their dimensions are still too large for on-chip integration. Hence, off-chip inductors are commonly employed to minimize the chip footprint [16,17].

In recent years, emerging concepts of spoof surface plasmons and skyrmions have unveiled novel strategies for attaining deep-subwavelength microwave resonances [18–22]. The concept of skyrmion was first proposed in the early 1960s as a topologically nontrivial soliton solution in nonlinear field theory [23,24], originally formulated to describe particles subject to strong interactions. The distinctive topological properties have extended the scope of the skyrmion concept, from condensed matter physics to the fields of optics and electromagnetics [25–30]. Similarly, the concept of surface plasmons has also been extended from the optical frequencies to microwave bands, mimicking their mode distributions and physical properties based on metamaterial structures [31–35]. Metamaterial design philosophy and planar circuit technologies provide substantial flexibility for developing the skyrmions and spoof surface plasmons in the microwave frequencies. Recently, skyrmion distributions have been realized in electric and magnetic resonances of spoof localized surface plasmons (SLSPs) [36–38], with an electrical size of 1/100 wavelength [36]. Benefiting from their compatibility with planar circuit processes and active devices, microwave sensing systems based on spoof plasmonic skyrmion resonators have been reported [39]. Utilizing high-precision integrated circuit (IC) technologies [40], more functional devices with much smaller electrical sizes can be expected with the spoof plasmonic skyrmion resonances as filtering networks.

**Figure 1.**
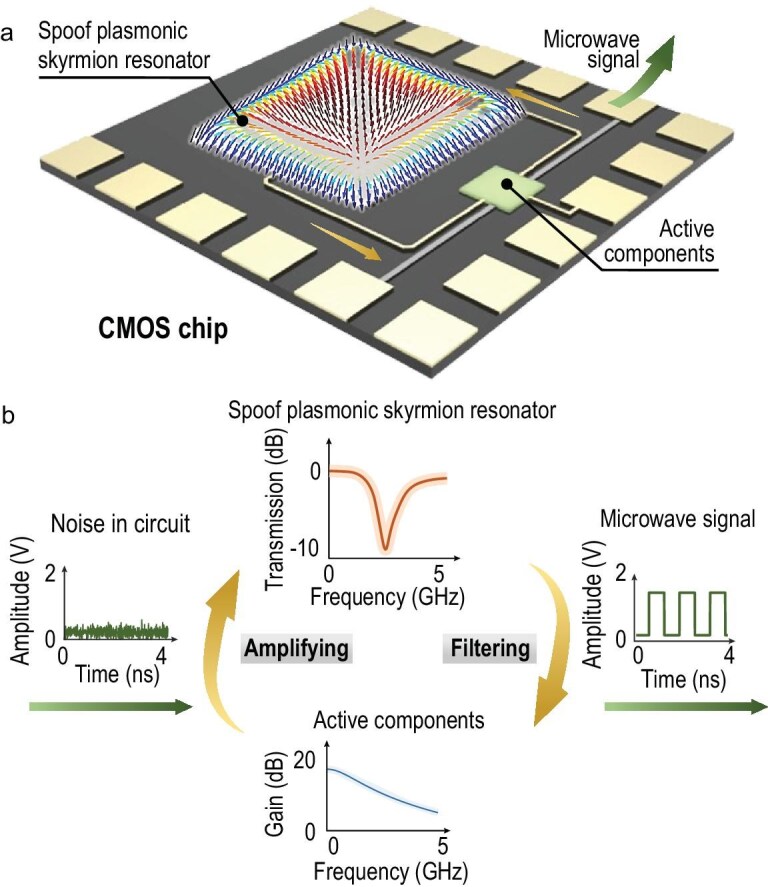
Conceptual illustration of the deep-subwavelength on-chip microwave oscillator based on the spoof plasmonic skyrmion resonator. (a) Schematic of the on-chip microwave oscillator. (b) The evolution of the signal in the oscillation circuit.

Here, we propose a deep-subwavelength on-chip microwave oscillator fabricated in a 0.18 μm complementary metal oxide semiconductor (CMOS) process, as illustrated in Fig. 1a. An SLSP resonator composed of square spiral lines in the top metal layer can confine the fundamental resonance into a deep-subwavelength scale and mimic the skyrmion field pattern. The primary challenge is to feed energy into this strongly confined mode efficiently. Hence, an interlayer excitation structure is designed to concentrate the electromagnetic power into the deep-subwavelength plasmonic skyrmion resonator, achieving an excitation efficiency of 83.6%. Moreover, the skyrmion resonator is innovatively incorporated into an active oscillator, establishing stable deep-subwavelength skyrmion oscillation fields. A complementary cross-coupled pair with high startup efficiency is designed to compensate for the high absorption loss. Figure 1b illustrates the microwave signal oscillation process, in which the active components amplify the tiny noise in the circuit, and the spoof plasmonic skyrmion resonator constitutes a positive feedback frequency-selecting network. The electric size of the core region (including both the active and passive components) reaches *λ*_0_/865, corresponding to a mode volume of 2.97 × 10^−11^ *λ*_0_^3^. This work presents the pioneering integration of EM skyrmions with active manipulations in a commercial integrated circuit process, promising significant advancements in skyrmion-based devices and chips.

## RESULTS AND DISCUSSIONS

### Mode analysis of spoof plasmonic skyrmion resonator

**Figure 2.**
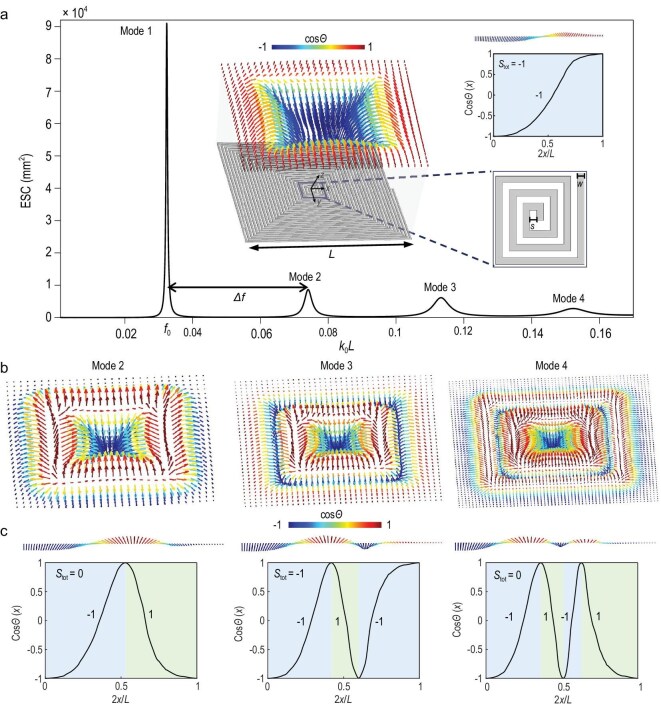
Mode analysis of the spoof plasmonic skyrmion resonator. (a) ECS of the spoof plasmonic skyrmion resonator. The insets show the resonator’s geometry, the distribution of the 3D magnetic field vector, the magnetic field vector configuration along the horizontal midline (*x* ≥ 0), and the zenith angle cosine distribution of the magnetic field vectors along the horizontal midline (*x* ≥ 0) for the fundamental mode. (b) The distributions of 3D magnetic field vectors for higher-order skyrmion resonances. (c) Magnetic field vector configurations (upper panel) and the zenith angle cosine distributions of the field vectors (lower panel) along the horizontal midline (*x* ≥ 0) for higher order modes. The vector length represents the magnitude of the local magnetic field.

Figure 2a illustrates the simulated extinction cross-section (ECS) spectrum of the spoof plasmonic skyrmion resonator. ECS is the sum of the scattering cross-section (SCS) and absorption cross-section (ACS). The result reveals four approximately equidistant resonance peaks with the frequencies of *f*_0_, *f*_0_ + *Δf, f*_0_ + 2*Δf* and *f*_0_ + 3*Δf*, respectively, where *f*_0_ is the fundamental resonance frequency, and *Δf* is the frequency spacing. Compared to spoof plasmonic skyrmion resonators implemented on PCB [36], the ECS of the micrometer-sized on-chip resonator decreases more rapidly as the mode number increases, indicating greater difficulty in exciting higher-order resonances. This behavior stems from higher material loss (i.e. ohmic loss) in the micrometer-scale on-chip patterns. Higher-order modes experience greater loss, resulting in a decrease in ECS and limiting their capability to interact with the external EM waves.

The insets in Fig. 2a show the structure of the spoof plasmonic skyrmion resonator, consisting of a tightly wound square spiral arm with 25 turns. Both width and pitch of the spiral arm are 3 μm (i.e. *w* = *s* = 3 μm). Additionally, the insets illustrate the three-dimensional (3D) vector magnetic field distribution of the fundamental mode, exhibiting a hedgehog-like pattern typical of a Néel-type skyrmion [41]. The topological properties of the skyrmion field distribution can be quantitatively evaluated by the skyrmion number *S* [42]:


(1) \begin{eqnarray*}
S = \frac{1}{{4\pi }}\int\!\!\int h \cdot \left( {\frac{{dh}}{{dx}} \cdot \frac{{dh}}{{dy}}} \right)\textit{dxdy},
\end{eqnarray*}

where *h* = {*H*_x_, *H*_y_, *H*_z_}/|*H*| is the local unit vector of the magnetic field, and the integrand represents the skyrmion density. The skyrmion number is a topological invariant to quantify the number of times the field wraps around a unit sphere. Since the cross-section of magnetic field is axisymmetric, the unit vector of the magnetic field can be written as *h*(*x, y, z*) = {sin*Θ*(*x*)cos*φ*, sin*Θ*(*x*)sin*φ*, cos*Θ*(*x*)} [43], where *x* is the distance from the center along the horizontal midline, *φ* is the azimuthal angle between the projection of the unit vector in the *xy*-plane and the *x*-axis, and *Θ*(*x*) is the zenith angle, the angle between the unit vector and the *z*-axis. The zenith angle of the unit vector varies by π along the horizontal midline, representing a lobe, and the *k*th mode has *k* lobes. The skyrmion number for the *i*th lobe of the mode profile along the horizontal midline direction of each mode is approximately equal to:


(2) \begin{eqnarray*}
{S}_i &=& \frac{1}{{4\pi }}\int_{0}^{{2\pi }}{{d\varphi \int_{{{x}_i}}^{{{x}_{i + 1}}}{{dx\frac{{d\Theta \left( x \right)}}{{dx}}}}}}\sin \Theta \left( x \right)\\ &=& - \frac{1}{2}\cos \Theta \left( x \right)\mathop |\nolimits_{{x}_i}^{{x}_{i + 1}} .
\end{eqnarray*}

Equation (2) shows that the skyrmion number depends only on the initial and final states of *Θ*(*x*). Each lobe of a mode profile has a skyrmion number of +1 or −1, representing an elementary skyrmion polarized in opposite directions. The inset in the top right of Fig. 2a demonstrates the cos*Θ*(*x*) distribution along the horizontal midline for the fundamental mode, corresponding to an elementary skyrmion with a total skyrmion number *S*_tot_ = −1. The *k*th-order mode corresponds to a kπ-skyrmion. The vector magnetic field distributions, the unit vector configuration of the magnetic field along the horizontal midline, and the distribution of the zenith angle cosine along the horizontal midline for *k* = 2, 3, 4 are showcased in Fig. 2b and c. The *k*th-order mode exhibits a kπ-twist field structure and controls the total skyrmion number between 0 and 1 (or −1).

### Excitation of the deep-subwavelength spoof plasmonic skyrmion resonator

**Figure 3.**
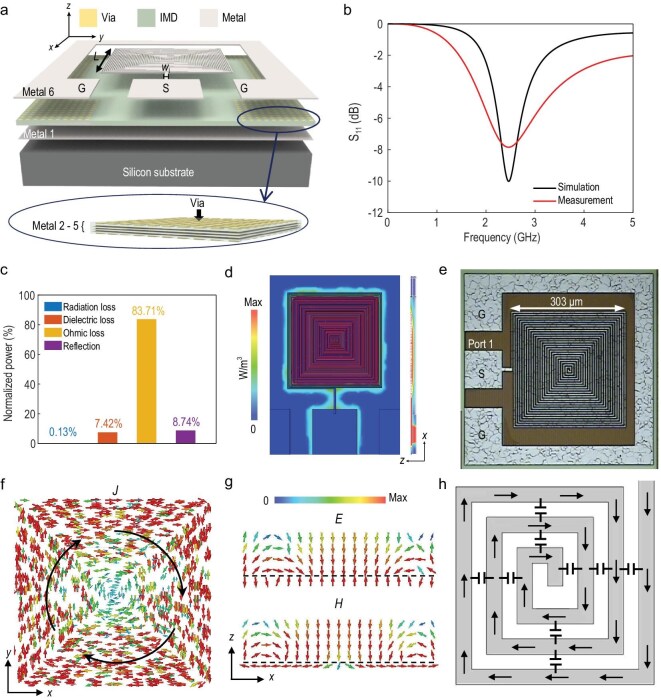
Properties of the spoof plasmonic skyrmion resonator fabricated using the 0.18 μm CMOS process. (a) The layout schematic. (b) Simulated and measured S_11_ of the resonator, in which the black and red lines represent the simulated and measured results, respectively. (c) Classification of the power loss of the resonator (normalized to the input power). (d) Simulated power-loss density of the resonator. (e) Microphotograph of the spoof plasmonic skyrmion resonator on the CMOS chip. (f) Simulated current distributions. (g) Simulated electric and magnetic field distributions on the *y* = 0 plane. (h) Schematic of the spiral structure with high equivalent capacitance.

Feeding EM energy into extremely deep-subwavelength sizes has always been challenging due to the diffraction limit. Here, a strong coupling excitation structure is proposed to enhance the coupling efficiency for the on-chip plasmonic skyrmion resonator. Figure 3a shows the layout of the spoof plasmonic skyrmion resonator based on the 0.18 μm CMOS process, which incorporates six metal layers separated by the intermetallic dielectric (IMD). Here, the purely passive plasmonic resonator is excited directly by a microstrip line, and the other end is connected to the ground-signal-ground (GSG) port. Both the resonator and the microstrip line are implemented on the top metal layer (Metal 6 layer), with a large area of metal in the Metal 1 layer serving as the ground. The edge length *L* of the entire resonator is 303 μm, with the width of the microstrip line being 9 μm. The simulated reflectance spectrum (S_11_) is demonstrated as the black line in Fig. 3b, showing a resonance frequency of 2.46 GHz, and an excitation efficiency of 90% with an S_11_ value of −10 dB. Figure 3c shows the distribution of power entering the resonator, where 8.74% of the input power is reflected through the port, while the remainder is dissipated internally as inherent loss in the resonator. High intrinsic loss, including radiation loss, dielectric loss, and ohmic loss, results in a low-quality factor. The simulated radiation efficiency of this on-chip resonator is −28.95 dB, indicating that only 0.13% of the power is radiated into free space. The simulated power loss density, including dielectric and ohmic losses, is illustrated in Fig. 3d. Although a portion of the EM field penetrates into the dielectric substrate, the dielectric loss accounts for only 7.42% of the input power. Due to the relatively low conductivity of the alloys used in the chip’s metal layers, the ohmic loss is substantial, reaching 83.71%. Additionally, the small thickness of the metal layers amplifies the impact of surface roughness, further increasing ohmic loss. The magnetic field skyrmion of the resonator originates from the circulating current illustrated in Fig. 3f, along the spiral metal line. The current vortices generate magnetic dipoles in the *z*-direction, and the magnetic field distributions exhibit a skyrmionic structure in the *xy*-plane. Figure 3g shows the electric and magnetic field distributions on the *y* = 0 plane.

A chip microphotograph of the fabricated spoof plasmonic skyrmion resonator is presented in Fig. 3e. Three pins of the GSG probe are pressed onto the corresponding pads on the left side of the chip to feed the excitation signal. The measured S_11_ is shown as a red line in Fig. 3b, illustrating a resonance frequency of 2.46 GHz and an excitation efficiency of 83.6% with an S_11_ value of −7.84 dB. Hence, the electrical size of the resonator equals 1/402.5*λ*_0_. The symmetric EM field distribution shown in Fig. S1 suppresses the radiation, concentrating the energy on the surface of the structure, which contributes to generation of strong localized fields. Furthermore, as presented in Fig. 3h, the parallel connection of multiple inter-turn capacitances yields a high equivalent capacitance for the spiral structure [44], enabling magnetic resonance at physical dimensions that are much smaller than the operating wavelength. The ultra-compact size of the spoof plasmonic resonator makes it ideal for high-density on-chip integration, while the topological property of the skyrmion makes it well-suited for on-chip fabrication with significant process variations [36].

### Principle of negative-resistance circuit

**Figure 4.**
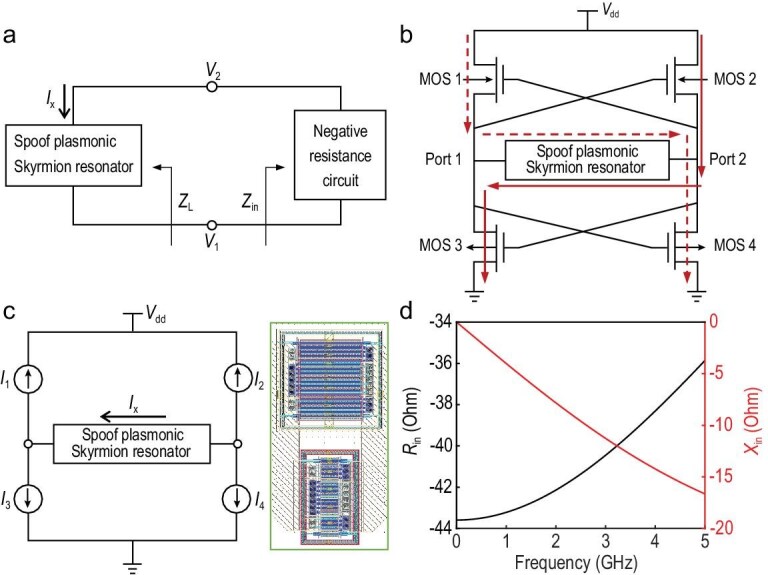
Principle of the negative resistance oscillator. (a) Circuit model of the negative resistance oscillator. (b) Schematic of the proposed plasmonic skyrmion oscillator based on the complementary cross-coupled pair. The solid red line and dashed red line represent the current paths during the positive and negative half-periods of the oscillation, respectively. (c) Small-signal model (left) and layout (right) for the MOSFET implementation. (d) Simulated resistance and reactance for the complementary cross-coupled pair.

The proposed microwave source is a self-excited oscillator based on negative resistance, consisting of a resonant unit and a negative-resistance circuit, as illustrated in Fig. 4a. The resonator simultaneously functions as a frequency selector and an energy storage element. A negative resistance circuit is required to compensate for the resonance loss and sustain stable oscillation. According to the Barkhausen criterion, the system must satisfy the following startup conditions to initiate the oscillation [45]:


(3) \begin{eqnarray*}
{R}_L + {R}_{\rm in} < 0,
\end{eqnarray*}

(4) \begin{eqnarray*}
{X}_L + {X}_{\rm in} = 0,
\end{eqnarray*}

where *R*_in_ is the effective negative resistance, and *R*_L_ represents the loss of the resonant unit. Equation (3) indicates that the loop gain is >1, while Equation (4) determines the oscillation frequency. This implies that even minute noise in the circuit can be amplified and filtered by the resonator to generate an oscillating signal at a specific frequency. As the oscillation amplitude grows continuously, it may lead to system instability. To prevent this, a cross-coupled transistor pair is typically used as a negative resistance component. The negative resistance provided by the cross-coupled pair enables a nonlinear amplitude stabilization mechanism: as the oscillation amplitude increases, the absolute value of negative resistance decreases. This nonlinear behavior ultimately ensures a steady-state oscillation amplitude, *V*_osc_. In the equilibrium state,


(5) \begin{eqnarray*}
{R}_L + {R}_{\rm in}\left( {{V}_{osc}} \right) = 0.
\end{eqnarray*}

Equations (4) and (5) together define the conditions for stable oscillation.

The deep-subwavelength on-chip spoof plasmonic skyrmion resonator exhibits a low Q-factor due to its inherently high loss [46], implying that the negative resistance component must compensate for greater energy dissipation to sustain oscillation. Thus, a complementary cross-coupled pair with enhanced startup efficiency is employed as the negative resistance circuit. The configuration of the complementary differential oscillator implemented using metal-oxide-semiconductor field effect transistors (MOSFETs) is shown in Fig. 4b. MOSFETs operating in the saturation region can be modeled as a voltage-controlled current source in parallel with a resistor [47]. The drain-to-source current is given by *I_i_* = *g*_m_*V*_GS_ (where *i* = 1, 2, 3, 4), where *g*_m_ denotes the transconductance of the MOSFET and *V*_GS_ is the gate-to-source voltage. Figure 4c presents the small-signal model of the MOSFET-based implementation, where the small parallel resistance is neglected for simplicity. Thus, the equivalent resistance is written as


(6) \begin{eqnarray*}
{R}_{\rm in} = \frac{{{V}_1 - {V}_2}}{{{I}_x}} = - \frac{2}{{{g}_{mN} + {g}_{mP}}},
\end{eqnarray*}

in which *g_mP_* and *g_mN_* are the transconductances of the P-metal oxide semiconductor (PMOS) transistors MOS 1 and MOS 2, and the N-metal oxide semiconductor (NMOS) transistors MOS 3 and MOS 4, respectively. The high efficiency of complementary cross-coupled pairs originates from the current reuse mechanism. The current paths are demonstrated in Fig. 4b. During each half oscillation period, the current flows through a pair of NMOS and PMOS transistors as well as the resonator. This indicates that the complementary cross-coupled pair delivers transconductance from both transistors simultaneously, enabling the entire energy to be effectively utilized to compensate for the resonator loss, as expressed in Equation (6). This configuration results in lower DC power consumption than the traditional NMOS or PMOS cross-coupled pairs, while offering the same negative resistance (see Note S1 for details). Furthermore, the complementary structure provides a larger oscillation amplitude and a more symmetrical output waveform, which enhances the phase noise performance [48].

Figure 4d shows the simulated impedance of the designed complementary cross-coupled pair. During the design process, the MOSFET size is carefully selected to achieve a sufficiently large negative resistance, thereby satisfying Equation (3) and leveraging the circuit instability to initiate oscillations. Considering the parasitic parameters of the actual device, a margin is typically incorporated to guarantee reliable oscillation startup. On the right side of Fig. 4c, the layout of the complementary cross-coupled pair is shown, where both PMOS and NMOS transistors are designed with multi-finger and multi-multiplier structures. The finger parameter represents the number of fingers in the interdigital gate, while the multiplier parameter defines the number of transistor repetitions. For transistors with large width-to-length ratios, a single gate is divided into a multi-finger structure to optimize the width-to-length ratio and minimize the parasitic effects and noise. Considering the flexibility of layout design, the multi-finger structure can be further divided into multiple identical transistors in parallel. This approach enables a more efficient transistor layout, ensuring optimal wiring and layout symmetry while further minimizing the parasitic effects. The PMOS transistors used in the complementary cross-coupled pair have a gate length of 0.18 μm and a gate width of 10 μm, with 4 fingers and a multiplier of 2. The NMOS transistors have a gate width of 3 μm, while all other parameters are consistent with the PMOS transistors. Additionally, guard rings [49] are placed around the transistors to prevent latch-up, isolate the noise, and reduce the impact of substrate parasitic resistance.

### Design and analysis of the deep-subwavelength oscillator

**Figure 5.**
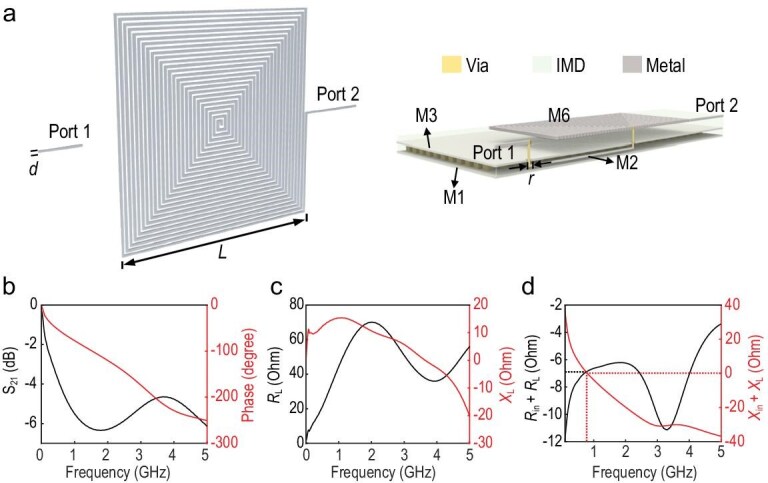
Design of the spoof plasmonic skyrmion resonator and analysis of the startup condition. (a) Schematic of the structure (left) and layout based on the CMOS process (right) for the spoof plasmonic skyrmion resonator. (b and c) Simulated S_21_ (b) and resistance and reactance (c) for the resonator. (d) Simulated resistance and reactance for the oscillator.

The passive plasmonic skyrmion resonator is integrated with active components to form the oscillator. The left panel of Fig. 5a shows the metal pattern in the Metal 6 layer, with the resonator structure identical to that in Fig. 2. A dual-port resonator required by the oscillator circuit is designed by an interlayer excitation structure. The sectional view in Fig. 5a illustrates the layout of this structure. A metal line in the Metal 2 layer is added to connect to the center of the resonator through a metal via-hole, forming an additional excitation port. A large area of metal in the Metal 3 layer is connected to the ground for isolation. Figure 5b and c demonstrates the transmission spectrum (S_21_) and equivalent impedance of the resonator (a detailed discussion on the loss and skyrmion mode of this resonator is presented in Note S2). Since the resonator is a passive structure, the resistance is >0. According to Equation (3), the startup condition for the system means that the total resistance must be negative (i.e. the loop gain is >1). The oscillation frequency generally lies in the passband of the resonator S_21_, and the stable oscillation frequency is determined by Equation (4). Figure 5d shows the total impedance of the oscillator, where the resistance and reactance around 810 MHz fit the startup conditions (Equations 3 and 4). In addition to the passive resonator and complementary cross-coupled pair, the complete oscillator also incorporates two output buffers. The output buffers convert the output signal into a square wave and isolate the resonator from external measurement equipment. This isolation prevents the external input impedance from affecting oscillation (see Note S3).

**Figure 6.**
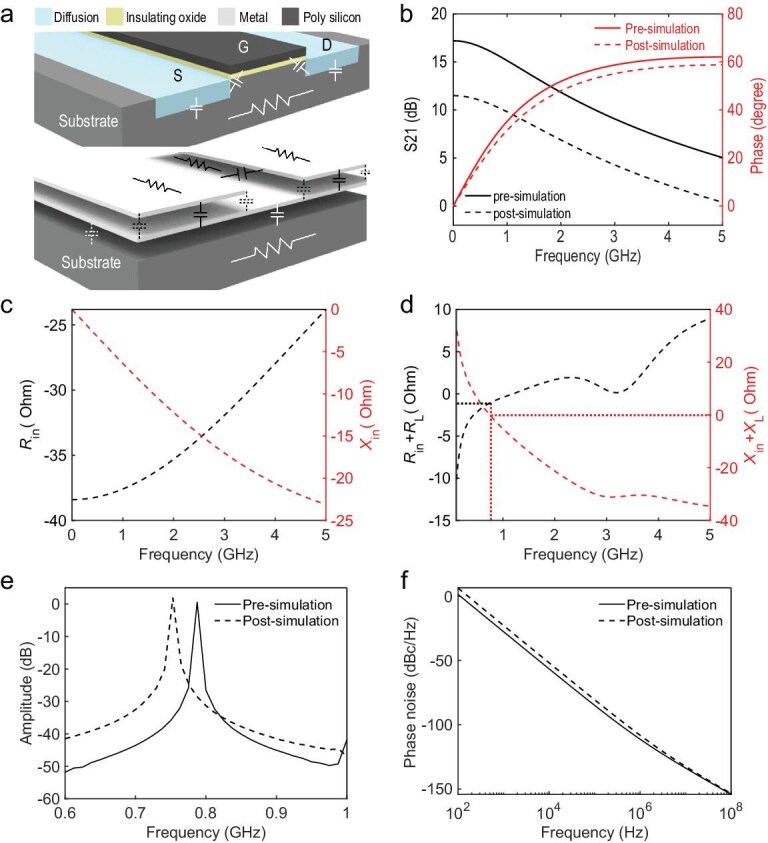
Pre- and post-simulation results of the oscillator. (a) Schematic of the parasitic parameters in the CMOS layout. (b) Pre- and post-simulated S_21_ of the active components. (c) Post-simulated resistance and reactance for the active components. (d) Post-simulated resistance and reactance for the oscillator. (e) Pre- and post-simulated output spectra. (f) Pre- and post-simulated phase noises.

In the IC design process, layout design and verification follow the determination of the schematic. The layout design ensures correctness and consistency through Design Rule Check and Layout Versus Schematic. After finalizing the layout, post-simulation is required to confirm that the chip’s electrical characteristics and timing constraints under actual operating conditions meet the design requirements. The first step in post-simulation is Parasitic Extraction, which typically extracts the parasitic capacitance and parasitic resistance of the circuit. Figure 6a shows the schematic of parasitic capacitance and resistance in the CMOS layout. The parasitic resistance mainly includes the resistance of the metal line, substrate resistance, source and drain diffusion resistance, and so on. The parasitic capacitance primarily includes the intermetallic capacitance and the capacitance between the transistor gate and source/drain. The next step is introducing these parasitic parameters into the schematic simulation to evaluate the impact of parasitic effects on circuit performance and signal integrity. Figure 6b and c illustrate the effects of parasitic capacitance and resistance on active components. The S_21_ amplitude of the active components decreases due to the parasitic resistances, while the S_21_ phase shifts as a result of parasitic capacitances. Correspondingly, the equivalent impedance *Z*_in_ of the active components changes compared to Fig. 4d, with the absolute value of the negative resistance decreasing.

The variation of impedance *Z*_in_ causes the startup conditions (Equations 3 and 4), which were satisfied in the pre-simulation, no longer to hold. The total resistance and reactance of the oscillator circuit are recalculated from the post-simulation results, as demonstrated in Fig. 6d. The results indicate that the startup conditions are satisfied at ∼750 MHz. The shift in oscillation frequency toward a lower value is due to the effect of parasitic capacitance. Figure 6e compares the pre-simulation and post-simulation results of the output spectrum. The post-simulated oscillation frequency is 752 MHz, which is lower than the oscillation frequency in the pre-simulation. Additionally, the parasitic parameters also affect the performance of the oscillator, such as causing a deterioration in phase noise. As illustrated in Fig. 6f, the phase noise obtained from post-simulation is slightly worse than that from pre-simulation. Based on the post-simulation result, the phase noise at the frequency offset of 1 MHz is −116.4 dBc/Hz.

**Figure 7.**
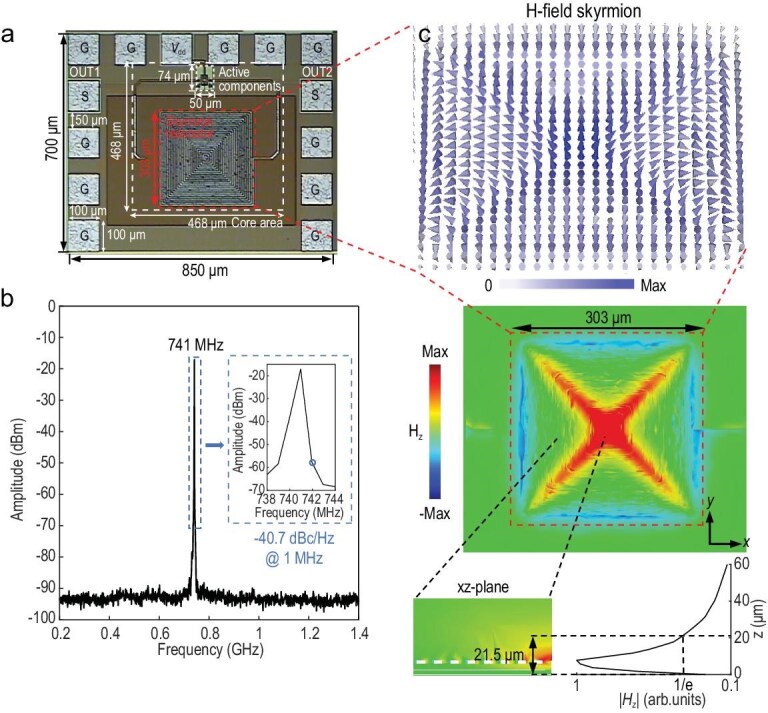
On-chip microwave source based on the 0.18 μm CMOS process. (a) Chip microphotograph of the proposed microwave source based on the spoof plasmonic skyrmion resonator. (b) Measured output spectrum of the proposed oscillator. The inset shows the enlarged spectrum around the peak. (c) Simulated vectorial magnetic field distribution (upper panel), and *H*_z_ profiles at the *xy*-plane and the *xz*-plane.

A microphotograph of the plasmonic skyrmion-based on-chip microwave oscillator is shown in Fig. 7a. The active components contain a complementary cross-coupled pair and output buffers, with the two differential ports connected to the two ports of the plasmonic skyrmion resonator (as shown in Fig. 5a). The third pad from the left at the top of the chip is used to access the 2 V DC voltage, the second pads from the top on the left and right sides are connected to the output ports of two output buffers, while all other pads are grounded. During measurement, a GSG probe is used to connect the output pad of the chip to the signal analyzer. The output spectrum measured by the signal analyzer is given in Fig. 7b. The oscillation frequency is ∼741 MHz, which is close to the oscillation frequency from post-simulation. The oscillation frequency falls in the P band. With excellent penetration and long-distance propagation capabilities, this frequency band can be applied in wireless communications, low-frequency Internet of Things (IoT), and so on [50,51]. In fact, the resonant and oscillation frequencies can be flexibly extended, as discussed in Note S5. The core area of the oscillator is 468 μm × 468 μm. The electrical sizes relative to the free-space wavelength *λ*_0_ and guided wavelength *λ*_g_ are *λ*_0_/865 and *λ*_g_/219.5, respectively (see Note S6 for details on the guided wavelength *λ*_g_). The measured output power is −17.11 dBm, and the phase noise at the frequency offset of 1 MHz is −40.7 dBc/Hz. Besides the inherent simulation errors, the main reason for this elevated phase noise is the mismatch of the output impedance and the standard 50 Ω. When the output port is connected to the signal analyzer via a 50 Ω probe, a significant reflection loss is induced. This issue can be solved by adding an output-matching network.

Figure 7c presents the simulated magnetic field of the active spoof plasmonic skyrmion oscillation mode, which exhibits a skyrmionic 3D vector field configuration. The EM wave behaves as a strongly evanescent wave in the *z*-direction [31]. Figure 7c shows the simulated *H*_z_ profile along the *z*-axis (the *z* = 0 plane corresponds to the position of the ground plane), with the field strength reaching the maximum at the surface of the Metal 6 metal. With a total decay length of 21.5 μm in both air and dielectric, the main energy of this oscillatory mode is concentrated in a volume of 303 × 303 × 21.5 μm^3^ (i.e. 2.97 × 10^−11^ *λ*_0_^3^ or 1.8 × 10^−9^ *λ*_g_^3^). A smaller mode volume indicates a higher concentration of the EM field (or energy), thereby enhancing the strength of wave-matter interactions at extreme scales. This characteristic makes the device promising for applications such as trace sensing and nonlinear effect enhancement [52].

## CONCLUSION

We report an on-chip microwave oscillator within a mode volume of 2.97 × 10^−11^ *λ*_0_^3^ and an electrical size of *λ*_0_/865. A spoof plasmonic skyrmion resonator realized by squared spiral metal lines in the 0.18 μm CMOS process is proposed to act as the deep-subwavelength filtering network. To efficiently concentrate the EM fields into the deep-subwavelength scale, a strongly coupled structure is introduced, and the high startup efficiency of a complementary cross-coupled pair is leveraged to compensate for the inherent high loss and low Q-factor of the skyrmion resonance. The deep-subwavelength on-chip oscillator holds great significance for pushing the frontiers of EM science at extreme scales. The integration of EM skyrmions with active circuit components is expected to accelerate research into skyrmion-based circuits and systems, enabling the discovery of novel physical phenomena and the development of next-generation integrated devices.

## Supplementary Material

nwag028_Supplemental_File

## References

[1] Gordon J, Zeiger H, Townes C. Molecular microwave oscillator and new hyperfine structure in the microwave spectrum of NH_3_. Phys Rev 1954; 95: 282–4. 10.1103/PhysRev.95.282

[2] Maiman T . Stimulated optical radiation in ruby. Nature 1960; 187: 493–4. 10.1038/187493a0

[3] Lin J, Farajollahi S, Fang Z et al. Electro-optic tuning of a single-frequency ultranarrow linewidth microdisk laser. Adv Photon 2022; 4: 036001. 10.1117/1.AP.4.3.036001

[4] Paul P, Toma E, Breger P et al. Observation of a train of attosecond pulses from high harmonic generation. Science 2001; 292**:** 1689–92. 10.1126/science.1059413 11387467

[5] Ma R, Luan H, Zhao Z et al. Twisted lattice nanocavity with theoretical quality factor exceeding 200 billion. Fundam Res 2023; 3: 537–43. 10.1016/j.fmre.2022.11.004 38933544 PMC11197626

[6] Ouyang Y, Luan H, Zhao Z et al. Singular dielectric nanolaser with atomic-scale field localization. Nature 2024; 632: 287–93. 10.1038/s41586-024-07674-9 39020170

[7] Ma H, Zhang Y, Zhou J et al. Infinitesimal optical singularity ruler for three-dimensional picometric metrology. Nat Commun 2024; 15: 10853. 10.1038/s41467-024-55210-0 39738042 PMC11686152

[8] Fei Z, Foley J, Gannett W et al. Ultraconfined plasmonic hotspots inside graphene nanobubbles. Nano Lett 2016; 16: 7842–8. 10.1021/acs.nanolett.6b04076 27960518

[9] Ma R, Oulton R, Sorger V et al. Plasmon lasers: coherent light source at molecular scales. Laser Photonics Rev 2013; 7: 1–21. 10.1002/lpor.201100040

[10] Barnes W, Dereux A, Ebbesen T. Surface plasmon subwavelength optics. Nature 2003; 424: 824–30. 10.1038/nature01937 12917696

[11] Noginov M, Zhu G, Belgrave A et al. Demonstration of a spaser-based nanolaser. Nature 2009; 460: 1110–2. 10.1038/nature08318 19684572

[12] Oulton R, Sorger V, Zentgraf T et al. Plasmon lasers at deep subwavelength scale. Nature 2009; 461: 629–32. 10.1038/nature08364 19718019

[13] Kwon S, Kang J, Seassal C et al. Subwavelength plasmonic lasing from a semiconductor nanodisk with silver nanopan cavity. Nano Lett 2010; 10: 3679–83. 10.1021/nl1021706 20704325

[14] Dong G, Shen Y, Hu S. On-chip localized surface plasmon resonator for 127 GHz compact CMOS oscillator. IEEE Electron Device Lett 2023; 44: 1927–30. 10.1109/LED.2023.3327106

[15] Dong G, Hu S, Shen Y. Mode-switchable localized surface plasmon resonator for W/D dual-band CMOS oscillator. IEEE Electron Device Lett 2024; 45: 762–5. 10.1109/LED.2024.3381157

[16] Blanco A, Rincón-Mora G. Compact fast-waking light/heat-harvesting 0.18-μm CMOS switched-inductor charger. IEEE Trans Circuits Syst I Regul Pap 2018; 65: 2024–34. 10.1109/TCSI.2017.2766188

[17] Tong C, Fan Z, Gao Y. A Li-ion battery input highly integrated LED driver with 96.8% peak efficiency and dual-color mixing capability. IEEE J Solid-State Circuits 2023; 59: 794–803. 10.1109/JSSC.2023.3331763

[18] Shen Y, Zhang Q, Shi P et al. Optical skyrmions and other topological quasiparticles of light. Nat Photonics 2023; 18: 15–25. 10.1038/s41566-023-01325-7

[19] Lin W, Ota Y, Arakawa Y et al. On-chip optical skyrmionic beam generators. Optica 2024; 11: 1588–94. 10.1364/OPTICA.540469

[20] Zhang X, Cui T. Deep-subwavelength and high-Q trapped mode induced by symmetry-broken in toroidal plasmonic resonator. IEEE Trans Antennas Propag 2021; 69: 2122–9. 10.1109/TAP.2020.3026480

[21] Zhang X, Cui T. Contactless glucose sensing at sub-micromole level using a deep-subwavelength decimeter-wave plasmonic resonator. Laser Photonics Rev 2022; 16: 2200221. 10.1002/lpor.202200221

[22] Bai T, Wang W, Zhang X et al. Exceptional point in a microwave plasmonic dipole resonator for sub-microliter solution sensing. Adv Funct Mater 2024; 34: 2312170. 10.1002/adfm.202312170

[23] Skyrme T . A non-linear field theory. Proc R Soc A 1961; 260: 127–38.

[24] Skyrme T . A unified field theory of mesons and baryons. J Nucl Phys 1962; 31: 556–69. 10.1016/0029-5582(62)90775-7

[25] Tsesses S, Ostrovsky E, Cohen K et al. Optical skyrmion lattice in evanescent electromagnetic fields. Science 2018; 361: 993–6. 10.1126/science.aau0227 30026318

[26] Du L, Yang A, Zayats V et al. Deep-subwavelength features of photonic skyrmions in a confined electromagnetic field with orbital angular momentum. Nat Phys 2019; 15: 650–4. 10.1038/s41567-019-0487-7

[27] Dai Y, Zhou Z, Ghosh A et al. Plasmonic topological quasiparticle on the nanometre and femtosecond scales. Nature 2020; 588: 616–9. 10.1038/s41586-020-3030-1 33361792

[28] Davis T, Janoschka D, Dreher P et al. Ultrafast vector imaging of plasmonic skyrmion dynamics with deep subwavelength resolution. Science 2020; 368: eaba6415. 10.1126/science.aba6415 32327571

[29] Shen Y, Hou Y, Papasimakis N et al. Supertoroidal light pulses as electromagnetic skyrmions propagating in free space. Nat Commun 2021; 12: 5891. 10.1038/s41467-021-26037-w 34625539 PMC8501108

[30] Yang A, Lei X, Shi P et al. Spin-manipulated photonic skyrmion-pair for pico-metric displacement sensing. Adv Sci (Weinh) 2023; 10: e2205249. 10.1002/advs.202205249 36840648 PMC10131799

[31] Pendry J, Martín-Moreno L, Garcia-Vidal F. Mimicking surface plasmons with structured surfaces. Science 2004; 305: 847–8. 10.1126/science.1098999 15247438

[32] Pors A, Moreno E, Martin-Moreno L et al. Localized spoof plasmons arise while texturing closed surfaces. Phys Rev Lett 2012; 108: 223905. 10.1103/PhysRevLett.108.223905 23003598

[33] Cui T, Zhang S, Alù A et al. Roadmap on electromagnetic metamaterials and metasurfaces. J Phys-Photon 2024; 6: 032502. 10.1088/2515-7647/ad1a3b

[34] Liu C, Yang F, Xu S et al. Reconfigurable metasurface: a systematic categorization and recent advances. EM Science 2023; 1: 0040021.

[35] Jung I, Peng Z, Rahmat-Samii Y. Recent advances in reconfigurable electromagnetic surfaces: engineering design, full-wave analysis, and large-scale optimization. EM Science 2024; 2: 0070201.

[36] Deng Z, Shi T, Krasnok A et al. Observation of localized magnetic plasmon skyrmions. Nat Commun 2022; 13: 8. 35013246 10.1038/s41467-021-27710-w PMC8748431

[37] Yang J, Zheng X, Wang J et al. Symmetry-protected spoof localized surface plasmonic skyrmion. Laser Photonics Rev 2022; 16: 2200007. 10.1002/lpor.202200007

[38] Li X, Liu L, Zhou Z et al. Highly sensitive and topologically robust multimode sensing on spoof plasmonic skyrmions. Adv Opt Mater 2022; 10: 2200331. 10.1002/adom.202200331

[39] Zhang X, Zhu J, Cui T. An ultracompact spoof surface plasmon sensing system for adaptive and accurate detection of gas using a smartphone. Eng 2023; 35: 86–94.

[40] Wu K, Rahman M. Pulse generation and compression techniques for microwave electronics and ultrafast systems. EM Science 2023; 1: 0010131.

[41] Kézsmárki I, Bordács S, Milde P et al. Neel-type skyrmion lattice with confined orientation in the polar magnetic semiconductor GaV_4_S_8_. Nat Mater 2015; 14: 1116–22. 26343913 10.1038/nmat4402

[42] Heinze S, von Bergmann K, Menzel M et al. Spontaneous atomic-scale magnetic skyrmion lattice in two dimensions. Nat Phys 2011; 7: 713–8. 10.1038/nphys2045

[43] Shen Y, Martínez E, Rosales-Guzmán C. Generation of optical skyrmions with tunable topological textures. ACS Photonics 2022; 9: 296–303. 10.1021/acsphotonics.1c01703

[44] Baena J, Marqués R, Medina F et al. Artificial magnetic metamaterial design by using spiral resonators. Phys Rev B 2004; 69: 014402. 10.1103/PhysRevB.69.014402

[45] Gonzalez G . Foundations of Oscillator Circuit Design. London: Artech House, Inc., 2006.

[46] Zhang G, Xing L, Xu Q et al. A frequency tunable liquid cavity bandpass filter. EM Science 2023; 1: 0020062.

[47] Sah C . Characteristics of the metal-oxide-semiconductor transistors. IEEE Trans Electron Devices 1964; 11: 324–45. 10.1109/T-ED.1964.15336

[48] Hajimiri A, Lee T. Design issues in CMOS differential LC oscillators. IEEE J Solid-State Circuits 1999; 34: 717–24. 10.1109/4.760384

[49] Voldman S, Perez C, Watson A. Guard rings: structures, design methodology, integration, experimental results, and analysis for RF CMOS and RF mixed signal BiCMOS silicon germanium technology. J Electrostat 2006; 64: 730–43. 10.1016/j.elstat.2006.05.006

[50] Oh K, Sankaran S, Wu H et al. Full-duplex crystalless CMOS transceiver with an on-chip antenna for wireless communication in a hybrid engine controller board. IEEE J Solid-State Circuits 2013; 48: 1327–42. 10.1109/JSSC.2013.2257479

[51] Qin R, You F, Xu J et al. A 0.12-nJ/b 20-mb/s 700-MHz GFSK transmitter with a subsampling PLL employing 8-bit class-D DCO. IEEE Microw Wirel Tech 2025; 35: 1081–4. 10.1109/LMWT.2025.3559352

[52] Schuller J, Barnard E, Cai W et al. Plasmonics for extreme light concentration and manipulation. Nat Mater 2010; 9: 193–204. 10.1038/nmat2630 20168343

