# Supplementary material for: On-chip microwave oscillator in deep-subwavelength scale based on spoof plasmonic skyrmions
Source: Natl Sci Rev. 2026 Jan 19;13(5):nwag028. doi: 10.1093/nsr/nwag028 (PMC12931317; doi:10.1093/nsr/nwag028)
Supplement: nwag028_Supplemental_File [file nwag028_supplemental_file.docx]

Supplementary Materials for

**On-chip microwave oscillator in deep-subwavelength scale based on spoof plasmonic skyrmions**

Wan Zhu Wang^1, 2^, Yu Liu^1^, Xuanru Zhang^1, 2,*^, Zhi Lin Gao^1, 2^, Xu Sheng Tang^3^, and Tie Jun Cui^1, 2,*^

^1^ State Key Laboratory of Millimeter Waves, Southeast University, Nanjing 210096, China

^2^ School of Information Science and Engineering, Southeast University, Nanjing 210096, China

^3^ School of Cyber Science and Engineering, Southeast University, Nanjing 210096, China

^*^E-mail: zhangxru@seu.edu.cn, tjcui@seu.edu.cn

**Contents**

I. Supplementary Notes

II. Figures

III. References

**I. Supplementary Notes**

**Supplementary Note 1. High efficiency of the complementary cross-coupled pair**

The high efficiency of the complementary cross-coupled pair originates from its current reuse mechanism. The specific current paths involved in the current reuse process are shown in Figure S2a. During the positive half-period of the oscillation signal (*V*_1_ at high electric potential, *V*_2_ at low electric potential), MOS 1 is conducted because *V*_2_ (the gate of MOS 1) is at a low electric potential. This pulls *V*_1_ (the gate of MOS 4) to a higher electric potential, causing MOS 4 to be conducted. The current flows from the power supply into MOS 1, exits from the drain of MOS 1, passes through the passive resonator, enters MOS 4, and flows into the ground from the source of MOS 4. Similarly, during the negative half-period of the oscillation signal, MOS 2 and MOS 3 are conducted, and the current flows through MOS 2, the resonator, and MOS 3 to the ground. The improved efficiency of the complementary cross-coupled pair is attributed to the following two main factors:

1. **Transconductance multiplication**. In each half-oscillation period, the complementary cross-coupled pair drives the two transistors to function in a coordinated manner, as illustrated in Figure S2a. The transconductances of the two transistors superimpose constructively, forming the multiplication effect. Based on the selected width-to-length ratio, the transistor transconductance *g*_m_ and *V*_gs_-*V*_th_ are approximately proportional (where *V*_th_ is the threshold voltage of the MOS transistor). Therefore, a lower bias voltage is needed for complementary cross-coupled pairs to provide the same negative resistance as NMOS or PMOS cross-coupled pairs.
2. **Minimized static power consumption**. Ideally, the complementary cross-coupled pair can convert nearly all DC power into AC energy and deliver it to the resonator. Since MOS 1 & MOS 3 and MOS 2 & MOS 4 form push-pull structures, they cannot conduct simultaneously. Thus the current path from MOS 1 to MOS 3 or from MOS 2 to MOS 4 does not exist. Figure S2a indicates that all of the current flows through the resonator to compensate for its loss. In contrast, NMOS or PMOS cross-coupled pairs can only convert a portion of the DC energy into AC energy and inject it into the resonator, while the remaining portion provides DC bias. For the NMOS cross-coupled pair (Figure S2b), a static current path exists in every half period but does not support the oscillation.

**Supplementary Note 2.** **Loss and skyrmion mode of the dual-port resonator**

Figure S3a shows the distribution of power loss density in the two-port resonator. Of the incident power, 50.09% is reflected, and 23.17% is transmitted. The remaining power is dissipated within the resonator. As illustrated in Figure S3b, internal loss comprises radiation loss, dielectric loss, and ohmic loss. These three types of loss account for 0.00074%, 0.19%, and 26.54% of the input power, respectively. Figure S3c displays the skyrmion field within the dual-port resonator. The corresponding skyrmion number in Figure S3d is calculated to be 0.97, indicating that the field distribution slightly deviates from a purely ideal skyrmion field.

**Supplementary Note 3. Design and analysis of the output buffer**

The active components of the complete oscillator circuit include two output buffers in addition to the complementary cross-coupled pair, as shown in Figure S4a. The input of each output buffer is connected to one of the two differential output ports of the cross-coupled pair, while the output is connected to the GSG port for measurement. Each output buffer features a self-biased inverter comprising a pair of NMOS and PMOS transistors. Figure S4b presents the layout of the output buffer, which utilizes multi-multiplier structures for the PMOS and NMOS transistors, similar to the cross-coupled pair, to reduce the parasitic effects. The PMOS transistors used in the output buffers have a gate length of 0.18 µm and a gate width of 4 µm, with a multiplier of 2. The NMOS transistors have a gate width of 2 µm, while all other parameters are consistent with the PMOS transistors. Additionally, guard rings are used to prevent the latch-up effect ^[S1]^. The transient simulation is performed in Cadence Virtuoso, and the proposed circuit successfully oscillates. The DC supply voltage *V*_dd_ is 2 V, with a power consumption of 19.86 mW. The output oscillation waveform, shown in Figure 6c, exhibits an oscillation frequency of 790 MHz. To further evaluate the impact of the output buffers, Figure S4d compares the output spectrum with and without the output buffers. Owing to the high input impedance and low output impedance of the inverter, the output buffers isolate the resonator from external measurement equipment, thereby preventing the external input impedance from influencing the oscillation. Further, the output buffers function as waveform shapers, improving the signal quality and stability by converting irregular waveforms into rectangular ones, as illustrated in Figure S4c. The inverter, functioning as a threshold control device ^[S2]^, also exhibits a certain noise tolerance, making the output waveform more resilient to the input noise.

**Supplementary Note 4. Generation and application of sine wave and square wave**

On-chip oscillators typically cannot generate the ideal sine waves directly, primarily for two reasons. Firstly, an unavoidable nonlinear process within the system, namely the amplitude- stabilization mechanism^[S3]^, causes the waveform distortion. Secondly, the low Q-factor of the on-chip resonator hinders effective suppression of unwanted frequency components, leading to an output waveform that departs from the ideal sine wave at the fundamental frequency. The generation of an ideal sine wave requires the introduction of extra filters. Non-ideal sine waves can be converted into square waves via output buffers, as described in **Supplementary Note 3**. This approach offers a simple design and minimizes the chip area.

Sine waves and square waves possess distinct characteristics that make them suitable for different applications. The sine waves provide a pure spectrum and higher energy efficiency, making them ideal for radio astronomy detection and the carrier waves in communication systems ^[S4]^. The square waves are compatible with the digital circuits, exhibit high noise immunity, and are robust against interference. They are commonly used for clock signals, control signals, and digital communications ^[S5]^.

**Supplementary Note 5. Design and measurement of a 2.403 GHz on-chip oscillator**

The resonant and oscillation frequencies can be flexibly tuned. Firstly, the resonant frequency of the passive resonator depends on the line width and the number of turns in the spiral structure. By tailoring these passive elements, the resonant frequency can be extended to the gigahertz range. Secondly, the oscillation frequency of active components is governed by the transistor size and bias voltage, which typically provide sufficient negative resistance over a defined bandwidth.

Figure S5a illustrates the microphotograph of the plasmonic skyrmion-based on-chip microwave oscillator. The active components include a complementary cross-coupled pair and output buffers, similar to the design presented in the main text. The passive resonator consists of two square spiral arms, each with a metal wire width and inter-arm spacing of 6 μm. Ports extending from the two spiral arms are connected to the differential ports of the complementary cross-coupled pair, forming the oscillation loop. The third pad from the left on the top edge of the chip supplies a 2V DC voltage, while the second pads from the top on the left and right sides connect to the output ports of the two output buffers; all remaining pads are grounded. During measurement, a GSG probe connects the output pad to the signal analyzer. Figure S5b shows the measured output spectrum, with an oscillation frequency of approximately 2.403 GHz. The measured output power is -36.6 dBm, and the phase noise at a 1 MHz offset is -30.3 dBc/Hz. The maximum operating frequency of the designed oscillation signal is determined by the transistor cut-off frequency in the integrated circuit process. In the 0.18-μm CMOS process, this frequency can reach approximately 30GHz ^[S6]^, while in the advanced technology nodes such as the 40-nm CMOS process, it can exceed 100GHz ^[S7, S8]^. The on-chip oscillators operating in the gigahertz range can support a wide range of potential applications, including the RF transceivers, automotive radar, and wireless sensing systems ^[S9–S11]^.

**Supplementary Note 6. Guided wavelength in the circuit**

The actual wavelength propagating in the circuit, known as the guided wavelength, is defined as *λ*_g_ = *λ*_0_/(*ε*_eff_)^1/2^, where *ε*_eff_ is the effective permittivity: *ε*_eff_ = (*β*/*k*_0_)^2^, *β* is the propagation constant of the microstrip line, and *k*_0_ is the wave number in vacuum. Hence, the effective permittivity depends on the dielectric substrate, circuit structure, and the corresponding electromagnetic mode. Figure S6 shows the effective permittivity extracted from the CST full-wave simulation, where the multilayer intermetallic-dielectric stack and the 3 μm-wide metal line are used in the CMOS process. The effective permittivity is 15.56 at the oscillation frequency of 741 MHz. Thus, the guided wavelength *λ*_g_ equals approximately *λ*_0_/3.94.

**II．Figures**


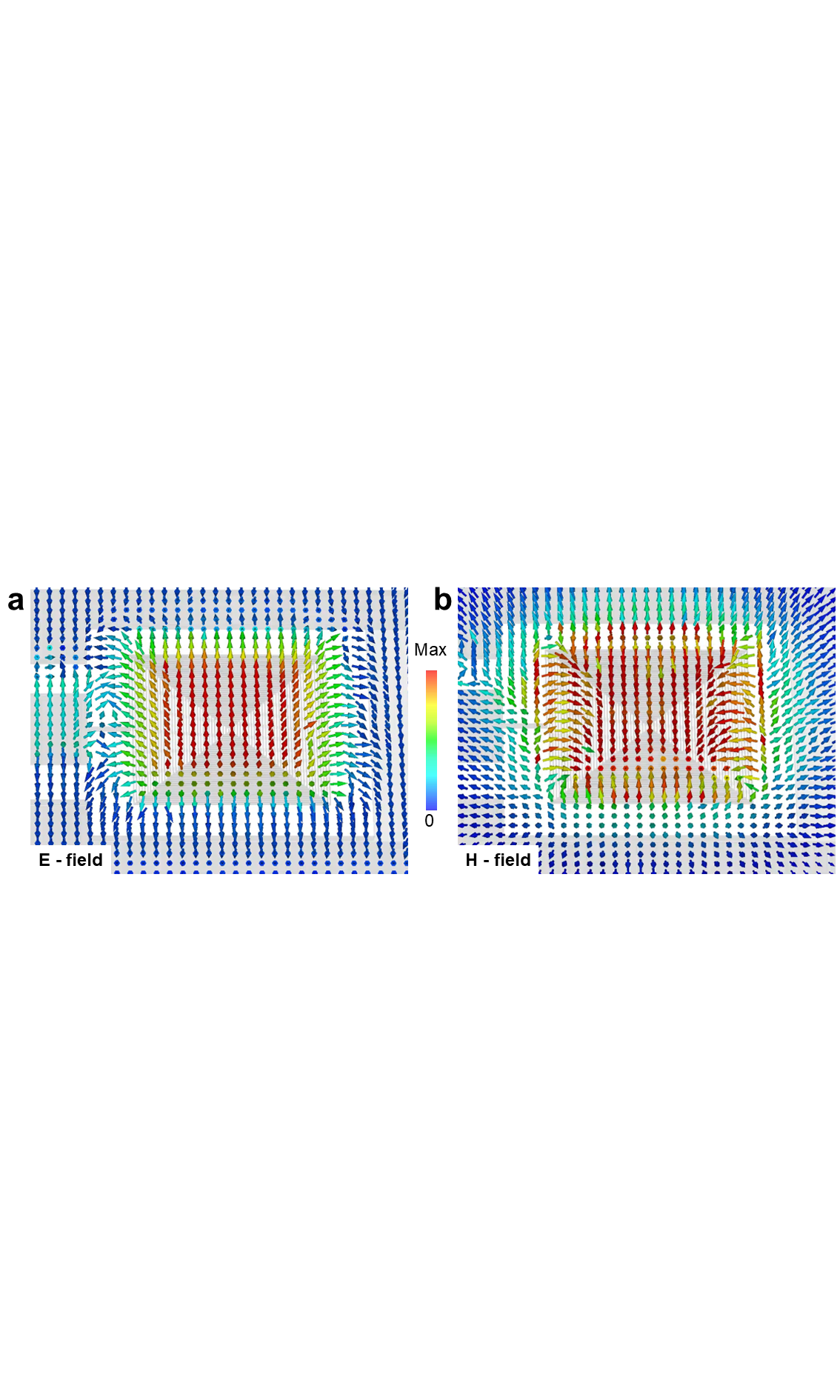


**Figure S1. Vector distributions of electric and magnetic fields.** (a) The E-field. (b) the H-field.


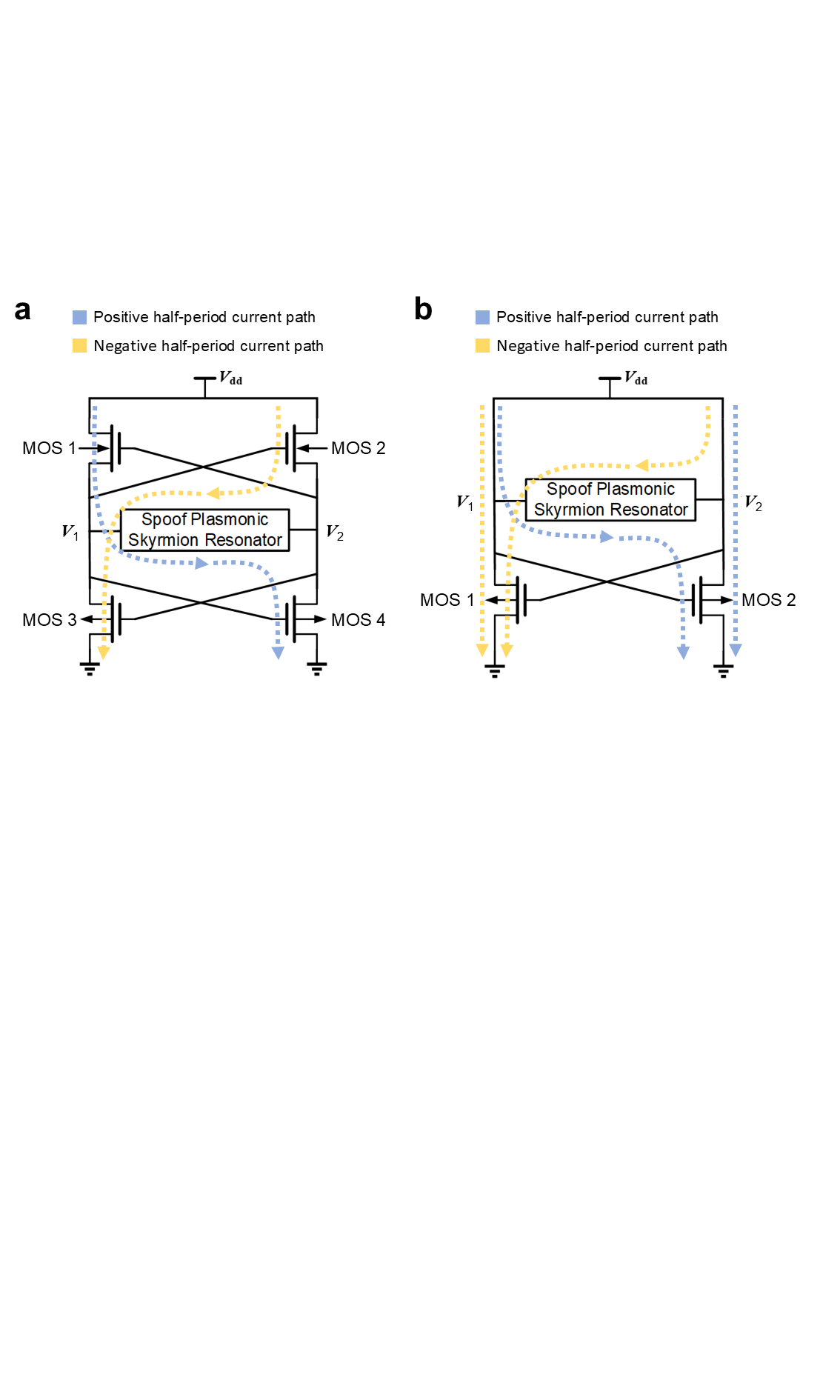


**Figure S2. Current paths for** the complementary cross-coupled pair (a) and the NMOS cross-coupled pair (b).


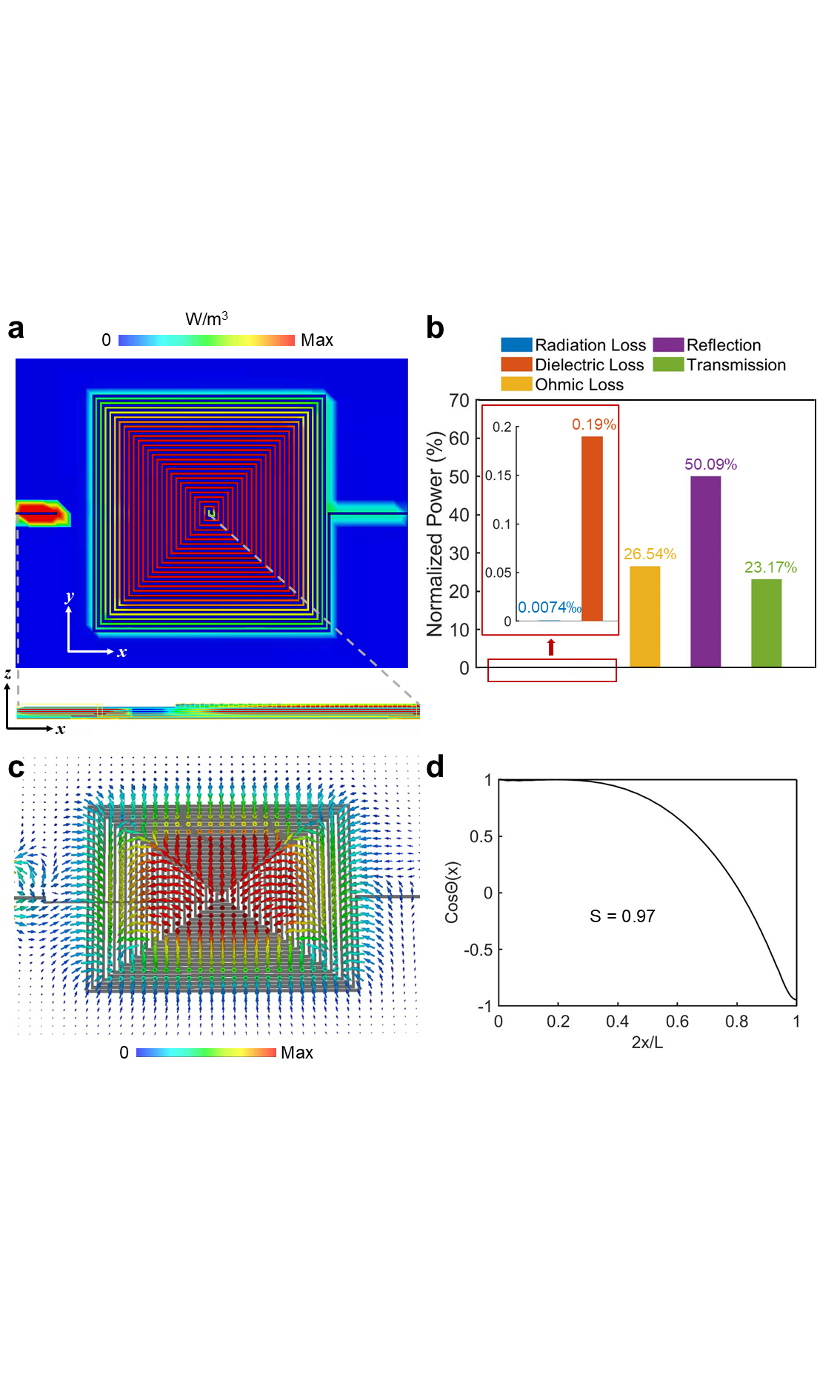


**Figure S3.** **Mode analysis of the dual-port plasmonic skyrmion resonator.** (a) Simulated power loss density of the dual-port resonator. (b) Classification of the dual-port resonator power loss (normalized to the input power). (c) The magnetic field distribution. (d) Calculated skyrmion number.


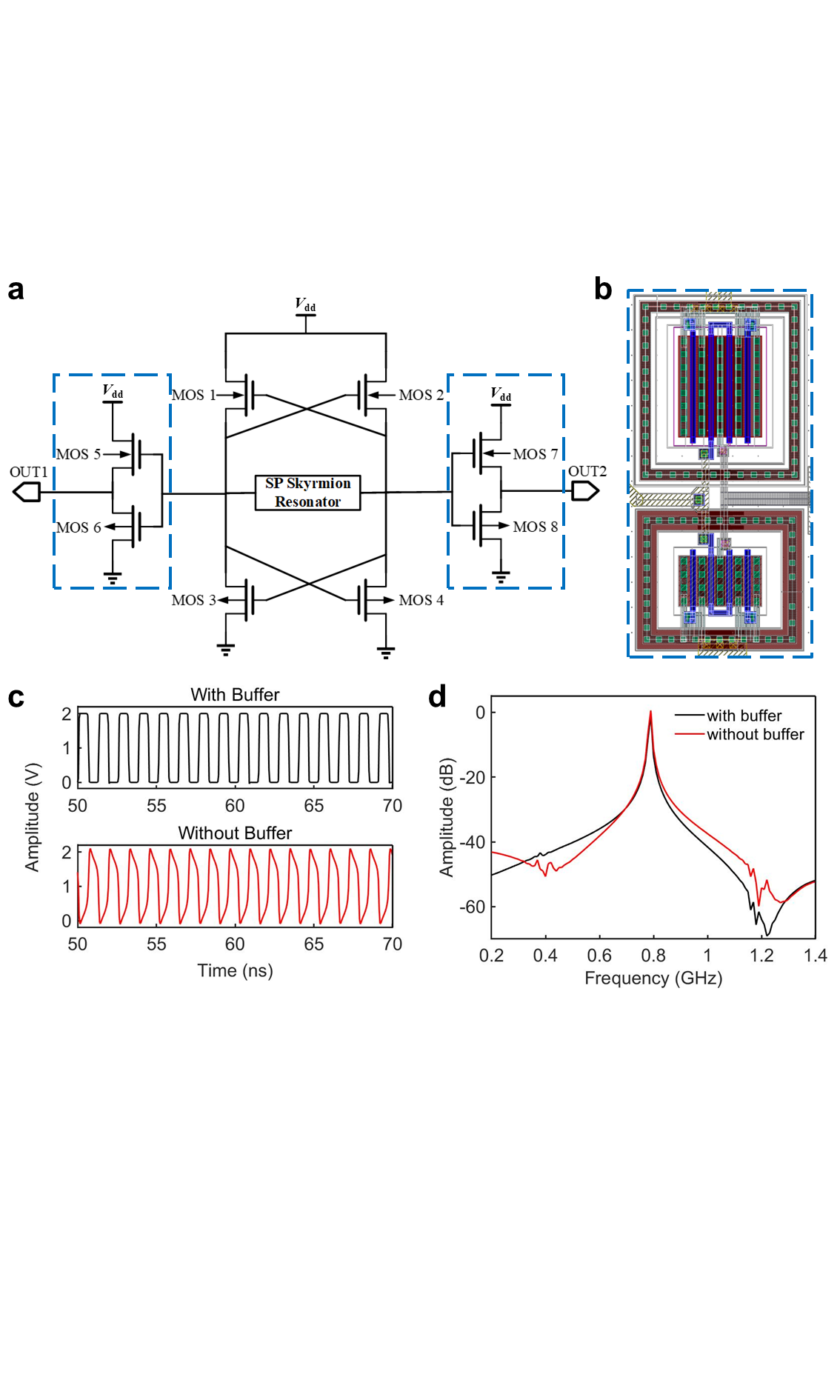


**Figure S4.** **Design and analysis of the output buffer.** (a) Schematic of the overall circuit. (b) The output buffer layout in the 0.18 CMOS process. (c) Simulated output waveform with/without the output buffers. (d) Simulated output spectra with/without the output buffers.


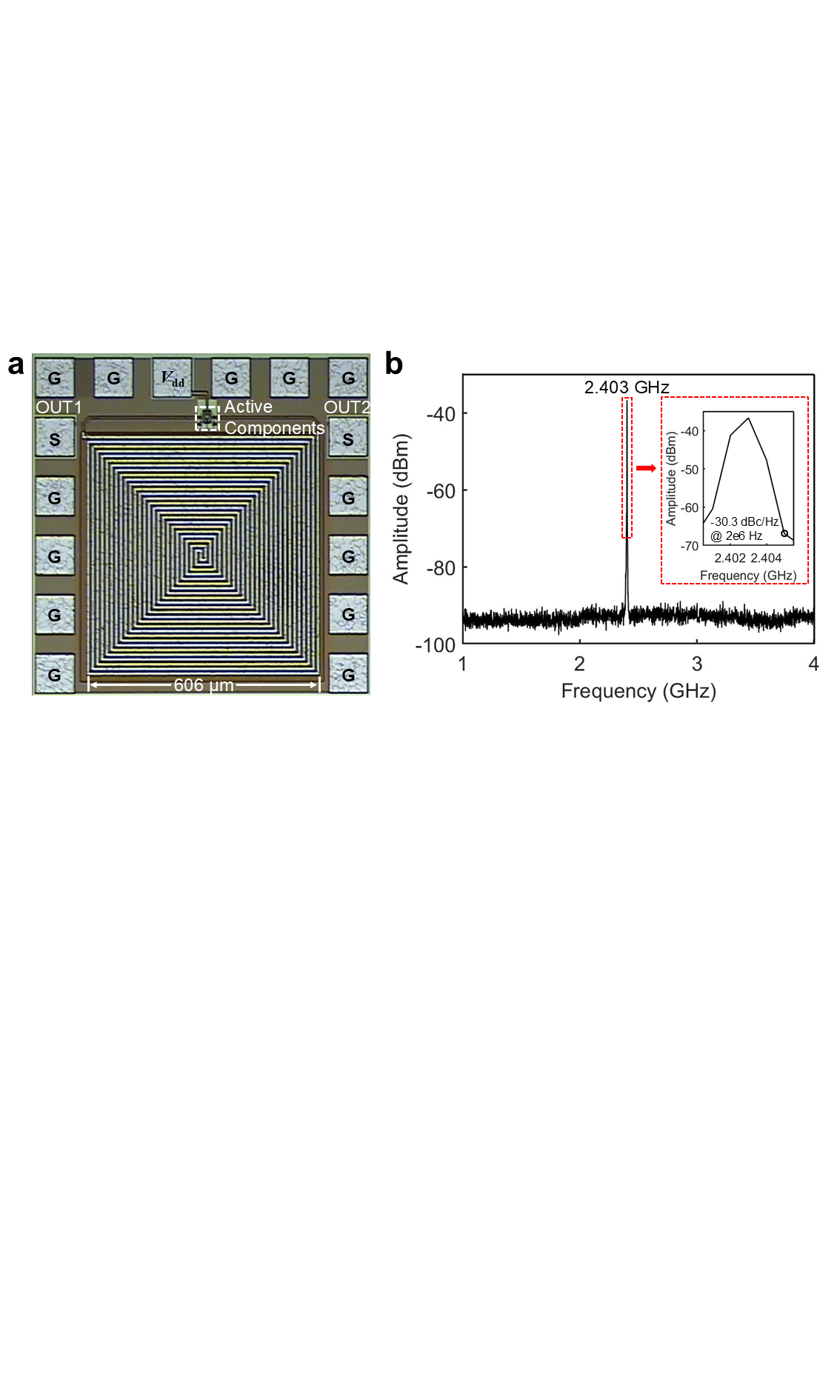


**Figure S5. A 2.403-GHz on-chip microwave oscillator.** (a) Chip micrograph of the 2.403-GHz source. (b) Measured output spectrum of the oscillator, with the inset showing an enlarged view around the peak.


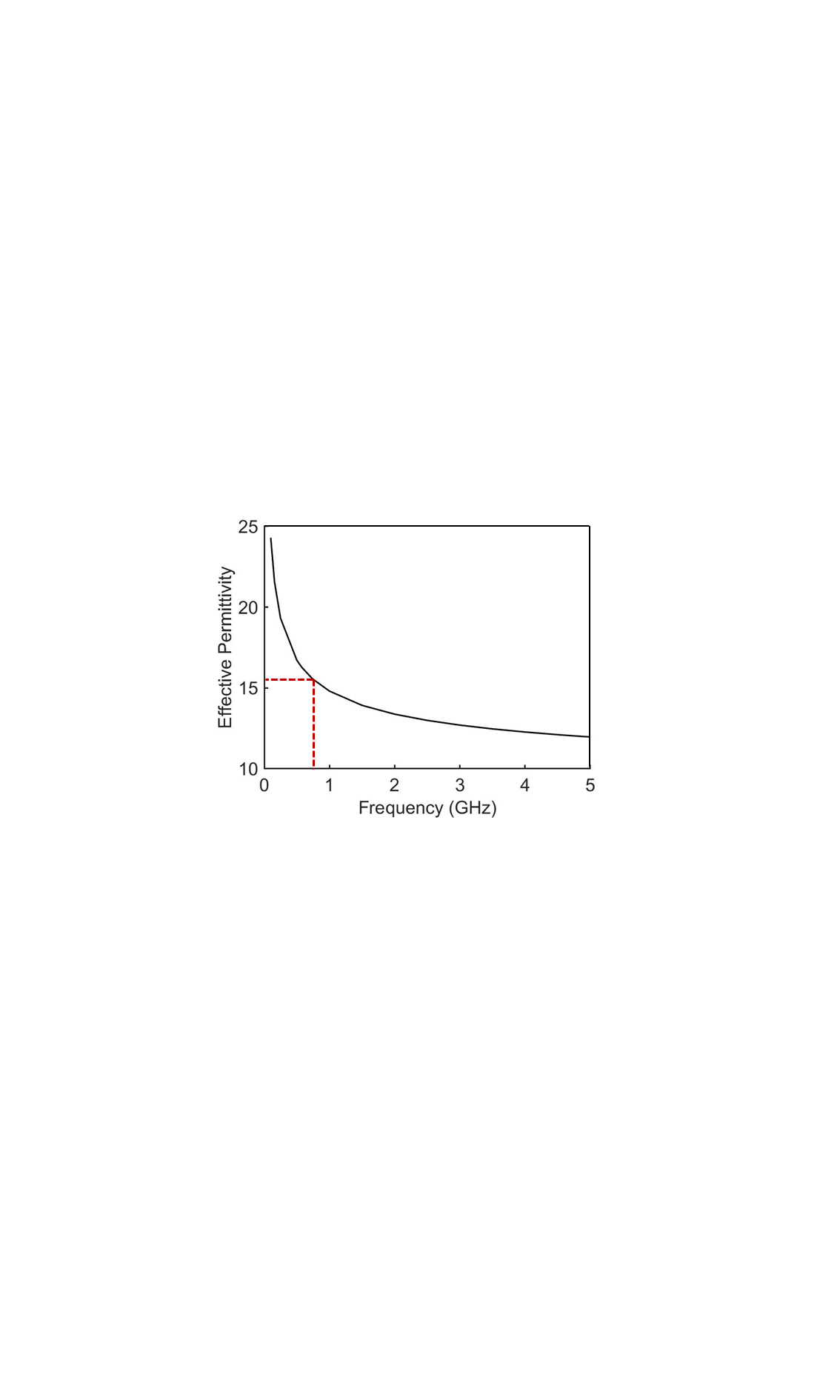


**Figure S6. Simulated effective permittivity of the proposed oscillator.**

**III. References**

[S1] Voldman S, Perez C and Watson A. Guard rings: Structures, design methodology, integration, experimental results, and analysis for RF CMOS and RF mixed signal BiCMOS silicon germanium technology. *J Electrostat* 2006; **64:** 730-43.

[S2] Rabaey J, Chandrakasan A and Nikolic B. *Digital Integrated Circuits: A Design Perspective*. Upper Saddle River, NJ: Pearson Education, 2003.

[S3] Lee T, and Hajimiri A. Oscillator phase noise: A tutorial. *IEEE J Solid-State Circuits* 2000; **35:** 326-36.

[S4] Kythe P. *Sinusoids: Theory and Technological Applications*. Boca Raton, FL: CRC Press, 2014.

[S5] Weber R. *Introduction to microwave circuits: Radio frequency and design applications*. New York, NY: John Wiley & Sons, 2001.

[S6] Kawahara K, Sawada J and Kamo T *et al.* Bandwidth tripler: Broadband signal generation with an image-rejection analog multiplexer for fiber optic transmitters. *IEEE T Microwave Theory* 2023; **71:** 22-34.

[S7] Dong G, Shen Y and Hu S. On-chip localized surface plasmon resonator for 127 GHz compact CMOS oscillator. *IEEE Electron Device Lett* 2023; **44:** 1927-30.

[S8] Dong G, Hu S and Shen Y. Mode-switchable localized surface plasmon resonator for W/D dual-band CMOS oscillator. *IEEE Electron Device Lett* 2024; **45:** 762-5.

[S9] Sene B, Reiter D and Knapp H *et al.* Design of a cost-efficient monostatic radar sensor with antenna on chip and lens in package. *IEEE T Microwave Theory* 2022; **70:** 502-12.

[S10] Li H, Rein H and Suttorp T. Fully integrated SiGe VCOs with powerful outinut buffer for 77-GHz automotive radar systems and applications around 100 GHz. *IEEE J Solid-State Circuits* 2004; **39:** 1650-8.

[S11] Sun Y and Babakhani A. Wirelessly-powered dielectric sensor with on-chip antennas in 180 nm SOI CMOS process. *IEEE Sens J* 2019; **19:** 2613-20.
